# Socio-demographic analysis of destination selection factors for Himalayan Hill destinations

**DOI:** 10.12688/f1000research.146873.2

**Published:** 2024-09-03

**Authors:** Manish Badoni, Babita Rawat, Megha Aggarwal

**Affiliations:** 1 USHHM, Uttaranchal University, Dehradun, Uttarakhand, India; 2 Uttaranchal Institute of Management, Uttaranchal University, Dehradun, Uttarakhand, India; 3 Uttaranchal Institute of Management, Uttaranchal University, Dehradun, Uttarakhand, India

**Keywords:** Himalayan Hill Destinations, Socio-demographic variables, Destination selection factors, Gender, Age, Occupation level, Income level

## Abstract

**Background:**

The towering peaks of the Himalayas lie in troves of captivating hill destinations, especially in India. Each destination aims to provide tourists with unique experiences and breath-taking landscapes. Understanding the tapestry of factors that weave the allure of these destinations and draw visitors from diverse backgrounds remains intriguing.

**Method:**

This study delves into the socio-demographic tapestry of Himalayan hill destination selection, unraveling the complex interplay of demographic characteristics, social influences, and individual motivations that shape tourists’ choices.

**Results:**

This study aims to answer why different tourists have different travel choices and what factors are the drivers behind such choices. The results show that destination selection factors are similar irrespective of respondents’ socio-demographic variabilities; however, for a few factors, the results are reversed.

**Conclusion:**

The study has implications for policymakers and the limitations of the research discussed at the end.

## Introduction

The Himalayan hill destinations of India have long captivated the imagination of travellers with their breathtaking landscapes, diverse cultures, and spiritual significance. The selection of factors that drive tourists to visit these destinations is crucial for both tourism practitioners and policymakers. Destination selection is a complex process influenced by a myriad of socio-demographic and travel motivation factors ( Kaushik *et al.*, 2010). This socio-demographic analysis of tourists with respect to destination selection factors provides valuable insights for tourism industry stakeholders. By recognizing the diverse preferences of different demographic groups, tourism practitioners can tailor their offerings to cater to a broad spectrum of tourists. This new understanding ensures that Himalayan hill destinations continue to attract visitors, while maintaining a delicate balance between economic development and environmental conservation.

Younger travellers are more likely to visit adventurous destinations, ( Chauhan & Jishtu, 2022) or older travellers would choose to relax to familiar and nearby destinations ( Wijaya *et al*., 2018). The higher income level group tended to travel to luxurious destinations and the other income level group would visit nearby destinations. These socio-demographic changes help customize marketing strategies to attract every class of tourists and increase footfall for a particular destination. Destinations with aesthetic images rightly communicated to tourists would be able to achieve a competitive advantage with respect to similar participative destinations. Understanding the intersection of sociodemographic and destination selection factors is vital. Related parties, such as destination marketers, tour operators, and policymakers, develop promotional campaigns, infrastructure, and tourism services based on these insights to ensure a more targeted and satisfying experience for tourists, contributing to the sustainable growth of the tourism industry ( Ma *et al*., 2018).

This study aims to construct a relationship between socio-demographic variables and destination selection factors for various destinations in Uttarakhand and Himachal Pradesh. It intends to highlight the sociodemographic factors that are majorly relevant for selecting a destination that further helps the destination to compete other similar geographical conditions. By examining the preferences and driving forces behind destination selection across various age groups, gender variability, occupation status, and income levels, this study aims to examine the nuanced decision-making processes that guide journeys to these major mountain havens.

## Literature review

The tourism industry is highly competitive, and the personal characteristics of individual tourists play an important role in destination selection. Even if destination attributes are unknown, certain individual traits motivate tourists to visit the place ( Suttikun *et al*., 2018). Motivation to visit a destination also plays a crucial role in shaping tourist behavior ( Baloglu & Uysal, 1996). Researchers have also identified push and pull factors as motivating factors for tourists to visit a destination, and pull factors serve as the basis for destination selection ( Josiam *et al*., 1999). Destination appeal is a major factor for tourists to visit the destination, whereas other factors such as infrastructure facilities, transportation availability, time, and cost involved in travel are secondary factors that enhance tourist flow ( Das *et al*., 2007). The study ( Hudson & Shephard, 1998) identified that not only adventure services but also travel information, accommodation, and tour operator services are selection factors for tourists. Other researchers ( Crompton, 1979; Dann, 1977, 1981) have also examined push and pull motivation factors in tourism. Push factors can be identified as knowledge of culture, status, personal development, relaxation, interpersonal relationships, and pleasure. On the other hand, pull factors could be atmosphere and climate at the destination, hygiene, built heritage, outdoor activities, and people’s characteristics ( Antara & Prameswari, 2018; Karamehmedović, 2018; Prayag & Ryan, 2011). Thus, the attributes or features of a destination are important factors for tourists.

Studies have been done in the past analyzed the relationship between the personal characteristics of tourists and their travel motivations and found that they are inter-related ( Chon, 1990; Court & Lupton, 1997; Joppe *et al*., 2001). The author ( Iyiola & Akintunde, 2011) examined whether tourist travel motivation to visit Nigeria is affected by sociodemographic variables. The motivation to select a destination can be determined by the age and income of tourists ( Ng *et al*., 2007; Yoon & Uysal, 2005). Travel motivating factors were also studied with respect to senior-age travellers, and the results are similar to those of previous studies with fewer pull factors, such as destination familiarity, value for money, and destination closeness ( Wijaya *et al*., 2018). Various authors ( Beerli & Martín, 2004; Um & Crompton, 1990; Walmsley & Jenkins, 1993) have studied the relationship between motivation to visit a destination and individual characteristics such as gender, age, education, occupation, and income as determinants of creating a destination image. Studies ( Gibson *et al*., 2008; Woodside & Lysonski, 1989) have also found similar results in that the perception of destinations is affected by tourist socio-demographic characteristics.

Tourist places are marketed according to the needs of potential tourists’ personal characteristics such as gender, age, education, occupation, and income ( Stabler, 2013; Um & Crompton, 1990). Sources such as advertising and word-of-mouth publicity provide travel inspiration to tourists, and past experiences play an important role in influencing future travel decisions for a potential destination ( Huang Songshan, 2006).

Various studies have analyzed the motivating factors and the influence of sociodemographic factors on creating destination image and tourist intention to visit. It is also possible that diverse visitors will have different levels of expectations with respect to different motivational factors and destinations. In this study, the influence of gender, age, occupation, and income on the expectation level of tourists pertaining to different tourist motivational factors, such as destination image, infrastructural facilities, beauty, culture, and heritage, is examined for selected destinations of Uttarakhand and Himachal Pradesh. The states of Uttarakhand and Himachal Pradesh have a lot of tourism potential, and both states are continuously putting a lot of marketing effort into creating an identity among tourists. Both the states in India have historical relevance in tourism and are attracting a lot of tourists towards different tourist destinations. The two states of India, Uttarakhand and Himachal Pradesh has been taken as study area because these states are comparable in terms of similar geography.

### Research objectives and hypothesis development

Based on the above literature review, this study aims to fulfill the following objectives and examine the related hypotheses:

Objective 1 To identify the selection factors to visit Himalayan region destination ( Antara & Prameswari, 2018; Das *et al.*, 2007; Karamehmedović, 2018; Prayag & Ryan, 2011).

Objective 2 To identify the relationship between sociodemographic variables and destination selection factors ( Beerli & Martín, 2004; Gibson *et al.*, 2008; Um & Crompton, 1990; Walmsley & Jenkins, 1993; Woodside & Lysonski, 1989).

Objective 2 is analyzed and fulfilled with the help of following related Hypothesis-

H1 Destination selection factors are significantly similar irrespective of the gender of the tourists

H2 Destination selection factors are significantly similar irrespective of the age of the tourists

H3 Destination selection factors are significantly similar irrespective of the occupation of the tourists

H4 Destination selection factors are significantly similar irrespective of the income of the tourists

## Methods

A self-administered questionnaire, prepared with a five-point Likert scale ( Likert, 1932) with 49 items, was prepared and distributed to Himalayan hill destinations of Uttarakhand and Himachal Pradesh, that is, Mussoorie, Shimla, Nainital, and Kangra, selected on the basis of convenience as these destinations are in close proximity. Experts from Academia and Tourism industry were approached for validating the questionnaire. Further pilot testing was performed on 100 responses and the value of Cronbach alpha was found to 0.897.

A total of 800 tourists were then approached, 200 at each destination, between April 2023 and November 2023. Out of 800, only 748 were included in the final analysis because Indian female, married, respondents were reluctant to complete the survey due to their reserved behaviour, and the survey was performed by their spouses or other relatives. Hence, these surveys were excluded from the final analysis. Although tourists were reluctant to participate in the survey, help from local restaurant owners, tour operators, and Mall Road vendors have been taken.

### Sociodemographic analysis

To fulfil the research objectives, responses collected from 748 tourists from selected Indian hill destinations were analyzed using a structured questionnaire. The respondents were selected based on their travel experience to a specific destination. Sociodemographic dimensions, such as gender, age, occupation, and income, were purposely included in the questionnaire to fulfil the research objectives. The distribution of the data is presented in Table 1.

**Table 1.** Respondents’ profile.

Age	n	%	Gender	n	%	Occupation	n	%	Income	n	%
Below 25 years	175	23.4	Male	531	71.0	Student	69	9.2	Below Rs.20,000	68	9.1
25-35 years	430	57.5	Female	217	29.0	Private sector	423	56.6	Rs.20,000 - Rs.50,000	423	56.6
35-45 years	116	15.5				Public sector	168	22.5	Rs.50,000 - Rs.80,000	165	22.1
Above 45 years	27	3.6				Business class	61	8.2	Rs.80,000 - Rs.1,00,000	65	8.7
						Other	27	3.6	Above than Rs.1,00,000	27	3.6
Total	748	100		748	100		748	100		748	100

Source: Author’s compilation.

Respondents’ sociodemographic analysis represented 57.5% of the youth tourist age ranging from to 25-35 years followed by 23% of respondents aged below 25 years. This indicates that about 80% of tourists belong to the young age group, that is, not more than 35 years. The maximum number of tourists was 71%, and 56% of the respondents belonged to private sector jobs, with incomes ranging from 20,000 Rs. to 50,000 Rs. Only 29% of the respondents were female as they were reluctant to participate in the survey. This behavior shows that the motivation of Indian females to travel to a destination still depends on their counterparts, whether their husbands, fathers, brothers, or friends.

Inferential Analysis

To identify the destination attributes or destination selection factors, a reliability analysis was performed, and the Cronbach’s alpha value of 0.909 showed that the questionnaire achieved high internal consistency. Before verifying the hypotheses set out in this study, an exploratory factorial analysis was conducted. The factors extracted by this method are uncorrelated and arranged in the order of decreasing variance. Bartlett’s test of sphericity and the calculation of Kaiser-Meyer-Olkin statistics indicate whether data are suitable for identifying orthogonal factor dimensions. Variables with loading equal to or greater than 0.4 were included in a given factor to decrease the probability of misclassification ( Hair *et al*., 1995). Forty-nine items were loaded saliently, and any factor that emerged with eigenvalues greater than one was considered for further analysis. The final factor distribution was allocated to forty-three items, other items with values below the threshold limit were not used for the final analysis. The total variance explained by factor analysis was 60%. The results of Cronbach’s alpha coefficients, KMO, Bartlett’s test of sphericity, and factor analysis are shown in Table 2.

**Table 2.** Results of factor analysis and factor distribution.

Factors	Item in the questionnaire	Item description	Factor loading	Cronbach’s alpha
Factor 1 (Destination Image & Attributes)	Q1	I try to cover the unique places of the destination	0.714	0.865
	Q3	I try to check with weather conditions before travel	0.604	
	Q4	I try to cover the least crowded places at the time of travel	0.819	
	Q5	I consider Local residents’ behaviour while selecting a place	0.739	
	Q7	I try to cover maximum popular places of the destinations	0.561	
	Q9	I look for places where telecommunication facilities are good	0.462	
	Q10	I look for market availability for local craft for gifting and souvenirs	0.648	
	Q11	I look for cleanliness at the destination while selecting	0.567	
	Q18	I travel at some special occasions like marriage anniversary or birthdays or new Year etc.	0.617	
	Q19	I look for language comfortability while selecting destinations	0.53	
	Q24	I look for Environmental condition of the destination	0.421	
Factor 2 (Value for Money)	Q34	I try to explore all the places you travel in limited time	0.602	0.763
	Q35	I try to find reasonable price for recreational activities	0.599	
	Q36	I look for travel time of the destination (3 days or 5 days or 7 days or more)	0.736	
	Q37	I would like to have political stability in destination	0.6	
	Q38	I seek for a place to relax with friends and family	0.73	
	Q43	I try to explore local cuisine and communicate with local people every time	0.448	
Factor 3 (Infrastructure Facilities)	Q6	I try to find diversity for accommodation while selecting the place to travel (Hotel/Homestay/villa/apartment/tent houses etc.)	0.557	0.735
	Q8	I try to check for local transport availability	0.49	
	Q13	I try to get accommodation with good view and space	0.688	
	Q14	I try to get accommodation with luxury facilities (like bathtub, shower, TV, refrigerator etc.)	0.753	
	Q20	I look for places where road administration is good	0.559	
Factor 4 (Beauty, Culture & Heritage)	Q26	Religious places play an important role while selecting the destination	0.701	0.770
	Q27	I try to cover all religious places of the destinations	0.625	
	Q28	I love to travel the places with scenic beauty and surroundings	0.701	
	Q29	I try to cover places with historic monuments	0.556	
Factor 5 (Tour & Travel Connections)	Q15	I rely on the information provided by tour operators	0.783	0.745
	Q16	I try to manage all your travel plans by your own	0.661	
	Q17	I try to go with good tour operators for travel plans	0.69	
Factor 6 (Value Added Services)	Q30	I look for recreational activities at the destination every time you travel	0.652	0.700
	Q31	I try to get good adventure facilities at the destination	0.77	
	Q32	I look for facilities like banks/ATMs while selecting a destination	0.47	
	Q33	I look for places where digital payments are upgraded for ease of payments	0.726	
Factor 7 (Destination Brand Value)	Q12	I try to cover the destinations showcase in movies and dramas (like Shimla in 3 idiots and Manali in Jab we met)	0.57	0.575
	Q21	I look for new and famous destinations every time you travel	0.564	
	Q25	I take feedback from your friends and colleagues who already travelled to the destination	0.63	
	Q39	I wait for right season to travel the place	0.6	
Factor 8 (Satisfaction and intention to Revisit)	Q44	I always feel satisfied with the quality of experiences in destination	0.558	0.817
	Q45	I am satisfied with the touristic attractions of the destination	0.614	
	Q46	I am satisfied with the infrastructure of the destination	0.787	
	Q47	I am satisfied with entertainment/recreational activities of the destination	0.702	
	Q48	I am satisfied with the culture/traditions of the destination	0.725	
	Q49	Overall, I am satisfied with the destination as a whole and revisit the destination	0.653	
Cronbach’s alpha of the total scale				0.909
Variance explained				60%
KMO				0.888
Bartlett				15095.16
Significance				.000

Source: Author’s compilation.

Table 2 shows the factors considered motivating and satisfactory for destination selection. Factors such as ‘Destination Image’, ‘Value for Money,’ ‘Infrastructure Facilities,’ ‘Beauty, Culture & Heritage’, ‘Tour & Travel Connections’, ‘Value added Services,’ Destination Brand Value’ are independent factors and able to create destination selection factor. The table also shows that the Cronbach alphas’ value reported on factor 7, i.e. ‘Destination Brand Value’ is low. This could be a consequence of this factor because the maximum number of respondents belonged to the income group of Rs. 20,000 to Rs. 50,000; for such respondents’ destinations, brand value would not be a motivating factor in selecting a destination. However, it was considered suitable to include this item because destination image would be helpful in creating destination brand value, and hence, becomes a motivating factor for the selection of a destination. The table also shows the dependent variable, destination satisfaction and intention to revisit.

The possible relationship between tourists’ socio-demographic characteristics and the selection factors of a destination was analyzed using ANOVA, checking its significance by means of the F statistic and p value (confidence level 95%).

The relationship between the sociodemographic variables of respondents and the factors of destination selection decisions of the respondents are shown in Table 3 with the help of ANOVA.

**Table 3.** Relationship between gender and destination selection factor.

Factors	Results	Sum of Squares	df	Mean Square	F	Sig.*
DI	Between Groups	5.967	1	5.967	0.118	0.732
	Within Groups	37786.443	746	50.652		
Vm	Between Groups	0.042	1	0.042	0.002	0.960
	Within Groups	12674.219	746	16.990		
Infra	Between Groups	0.243	1	0.243	0.019	0.891
	Within Groups	9648.708	746	12.934		
BeuCulHer	Between Groups	0.299	1	0.299	0.030	0.863
	Within Groups	7472.699	746	10.017		
TTC	Between Groups	2.921	1	2.921	0.499	0.480
	Within Groups	4365.485	746	5.852		
VaS	Between Groups	51.059	1	51.059	5.209	**0.023**
	Within Groups	7312.690	746	9.803		
BrandV	Between Groups	0.968	1	0.968	0.142	0.707
	Within Groups	5095.438	746	6.830		

Source: Author’s compilation.

The results show that there is a significant similarity between gender and destination selection factors, except for one factor, Value-added Services (VaS). This result justifies the fact that women tend to assess the value-added services of a destination more favorably than men. For all other factors, DI, Vm, Infra, BCH, TTC, and DBV, there were no significant differences irrespective of the gender of the respondents. Therefore, we confirm hypothesis H1 that Destination selection factors are significantly similar irrespective of the gender of tourists.

Table 4 shows the relationship between the age of respondents and destination selection factors. The results show that there is a significant difference among the selection factors for different age groups of the respondents.

**Table 4.** Relationship between age and destination selection factor.

Factors	Results	Sum of Squares	df	Mean Square	F	Sig.*
DI	Between Groups	3337.991	3	1112.664	24.027	0.000
	Within Groups	34454.419	744	46.310		
Vm	Between Groups	127.647	3	42.549	2.523	0.05
	Within Groups	12546.614	744	16.864		
Infra	Between Groups	58.686	3	19.562	1.518	0.209
	Within Groups	9590.266	744	12.890		
BeuCulHer	Between Groups	69.595	3	23.198	2.331	0.073
	Within Groups	7403.403	744	9.951		
TTC	Between Groups	145.140	3	48.380	8.523	0.000
	Within Groups	4223.267	744	5.676		
VaS	Between Groups	61.092	3	20.364	2.075	0.102
	Within Groups	7302.657	744	9.815		
BrandV	Between Groups	157.748	3	52.583	7.921	0.000
	Within Groups	4938.658	744	6.638		

Source: Author’s compilation.

Factors such as destination image, value for money, tour and travel connections, and destination brand value are considered differently by different age groups of tourists. For other factors, including infrastructure facilities, ‘Beauty, Culture and Heritage’, and ‘Value-added Services’, the results show no significant difference with the age of the respondents. Results implies that the selection factor ‘destination image’ or ‘Destination Brand Value’ are significantly different for different age categories of the tourists. Young tourists tend to be motivated by destinations with a grand brand value rather than older tourists. Similarly, a destination would have different age tourist footfall due to the difference in value for money. On the basis of these results, we partially confirm hypothesis H2 that Destination selection factors are significantly similar irrespective of the age of the tourists but only for factors ‘Infrastructure facilities’, ‘Beauty, Culture and Heritage,’ and ‘Value-added Services’.

Table 5 shows the result of occupation and reveals a significant difference between occupation and selection factors for destination, but only for the factors ‘Destination Image’, and ‘Value for Money’. The results reveal similarities among various occupation respondents and their selection factors for destinations, such as ‘Beauty, Culture & Heritage’, ‘Infrastructure facilities’, ‘tour & travel connections’, ‘value-added services’, and ‘destination brand value’. Results implies that the factor ‘Destination Image’ or ‘Value for money’ are significantly different for various occupational categories of the tourists. Tourists who are private sector employees motivate destinations that provide more value for money than other tourists. These results confirm hypothesis H3 that destination selection factors are significantly similar, irrespective of the occupation of the tourists.

**Table 5.** Relationship between occupation and destination selection factor.

Factors	Results	Sum of Squares	df	Mean Square	F	Sig.*
DI	Between Groups	1226.309	4	306.577	6.229	**0.000**
	Within Groups	36566.101	743	49.214		
Vm	Between Groups	176.014	4	44.003	2.616	**0.034**
	Within Groups	12498.247	743	16.821		
Infra	Between Groups	58.271	4	14.568	1.129	0.342
	Within Groups	9590.681	743	12.908		
BeuCulHer	Between Groups	90.143	4	22.536	2.268	0.060
	Within Groups	7382.856	743	9.937		
TTC	Between Groups	38.859	4	9.715	1.667	0.156
	Within Groups	4329.547	743	5.827		
VaS	Between Groups	33.237	4	8.309	0.842	0.499
	Within Groups	7330.511	743	9.866		
BrandV	Between Groups	61.639	4	15.410	2.274	0.060
	Within Groups	5034.767	743	6.776		

Source: Author’s compilation.

The relationship between the income of the respondents and the selection factors of the destination is shown in Table 6. The result shows there is no similarity between income and selection factors for destination only for the factors ‘Destination Image,’ ‘Value for Money,’ ‘Beauty, Culture & Heritage’. For other factors, ‘Infrastructure facilities,’ ‘Tour-Travel Connections’, ‘Value-added Services,’ and ‘Destination Brand Value’ showed significant similarity with the diverse income of respondents. Results implies that the factor ‘destination image’ or ‘Value for money’ are significantly different for different income categories of the tourists. Tourists with middle-income levels motivate and select destinations that provide more value for money than do other tourists. Thus, tourists with various income groups look for value for money and varied destination images rather than other destination selection criteria. These results partially confirm hypothesis H4 Destination selection factors are significantly similar irrespective of tourists’ income.

**Table 6.** Relationship between income and destination selection factor.

Factors	Results	Sum of Squares	df	Mean Square	F	Sig.*
DI	Between Groups	1282.551	4	320.638	6.525	**0.000**
	Within Groups	36509.859	743	49.138		
Vm	Between Groups	289.294	4	72.324	4.339	**0.002**
	Within Groups	12384.966	743	16.669		
Infra	Between Groups	45.355	4	11.339	0.877	0.477
	Within Groups	9603.597	743	12.925		
BeuCulHer	Between Groups	129.524	4	32.381	3.276	**0.011**
	Within Groups	7343.475	743	9.884		
TTC	Between Groups	43.114	4	10.779	1.852	0.117
	Within Groups	4325.292	743	5.821		
VaS	Between Groups	38.944	4	9.736	0.988	0.413
	Within Groups	7324.805	743	9.858		
BrandV	Between Groups	48.806	4	12.202	1.796	0.128
	Within Groups	5047.600	743	6.794		

Source: Author’s compilation.

## Results and discussion

This research was undertaken to identify various destination selection factors and their relationship with socio-demographic variables of tourists for various Himalayan region destinations in Uttarakhand and Himachal Pradesh, India. Various destination factors have been identified with the help of factor analysis, such as ‘Destination Image’, ‘Value for Money’, ‘Infrastructure facilities’, ‘Beauty, Culture & Heritage’, ‘Tour & Travel connections’, ‘value-added services, and ‘destination brand value’. These results are similar to previous studies such as Ng *et al.*, 2007; Wijaya *et al.*, 2018; Yoon & Uysal, 2005. Among all the above factors, ‘Destination image’ is a key determinant for travelers to visit any destination ( Kala, 2021). The Cronbach’s alpha value was 0.9, and the variance explained by all factors was 60%, which is considerably good ( Aggarwal *et al.*, 2024).

To further analyze the relationship between the factors and sociodemographic variables, one-way analysis of variance (ANOVA) was performed. The summary results of the ANOVA are presented in Table 7. The results are somewhat different as compare to previous studies in context to Indian destinations. The selection of destinations is independent with respect to socio‐demographic variables.Destination Image and Value for Money were found to be significantly different for different age groups, occupation status, and income levels. This implies that tourists of different age groups, occupation statuses, and income levels will have different choices for selecting a destination on the basis of ‘Destination image’ or ‘Value for money’. ‘Value added services’ was found dissimilar for socio-demographic variable ‘Gender’. This implies that the selection of a destination may change if value-added services are different for any destination. However, for other factors such as ‘destination image’ or ‘beauty, culture, and heritage’, the selection factor does not create any difference between male and female tourists.

**Table 7.** Comparative analysis of ANOVA results.

Sociodemographic factors/Motivational factors	Results	Destination image	Value for money	Infrastructure facilities	Beauty, Culture & Heritage	Tour & Travel Connections	Value added services	Destination brand value
**Gender**	F	0.118	0.002	0.019	0.030	0.499	5.209	0.142
	Sig.*	0.732	0.960	0.891	0.863	0.480	0.023*	0.707
**Age**	F	24.027	2.523	1.518	2.331	8.523	2.075	7.921
	Sig.*	0.000*	0.05*	0.209	0.073	0.000*	0.102	0.000*
**Occupation**	F	6.229	2.616	1.129	2.268	1.667	0.842	2.274
	Sig.*	0.000*	0.034*	0.342	0.060	0.156	0.499	0.060
**Income**	F	6.525	4.339	0.877	3.276	1.852	0.988	1.796
	Sig.*	0.000*	0.002*	0.477	0.011*	0.117	0.413	0.128

Source: Author’s compilation.

### Implications and Limitations

Destination selection factors are important for any destination to improve tourist footfall and gain a competitive advantage. Socio-demographic variables play an important role in such decisions and hence need to be taken care of by the DMOs. Destination marketing organizations should identify their tourists and their choices. Any tourist, if impacted by their socio-demographic variable, will try to select destination suits for their personality and pocket. In this study, results revealed that age and income are major contributing socio- demographic variables in case of destination selection for Indian destinations. ‘Destination Image’ and ‘Value for Money’ factors are impacted by different age group and income level of the tourist. Young tourists would love to visit Goa, whereas senior-aged tourists would like to travel to a destination with religious beliefs. Thus, destination marketing organizations should analyze their tourist choices and market their products according to the needs of tourists.

This research also has some limitations. The first and foremost limitation of this study is the selection of socio-demographic variables. This research used only four socio-demographic variables: Gender, Age, Occupation and Income. Other sociodemographic variables were beyond the scope of this study. The results may vary if other variables are included in future studies. The other limitation is the study area, which is related to the Himalayan region and included a few destinations of Uttarakhand and Himachal Pradesh with random selection. Future studies should include other destinations. A comparison between these destinations could also be performed with respect to sociodemographic variables. This study covers the Himalayan region, and other hill regions of India can also be studied with the help of similar analysis, and further comparisons can be drawn.

## Ethical statement

This research was conducted in accordance with the guidelines of the **Research Ethics Board** (REB) of **Uttaranchal University**. The Research Ethics Board has given the approval on March 4, 2023, and the approval number is UU/DRI/EC/2023/002.

The questionnaire has been submitted to REB of the university, the board members and chairperson have identified the viability of the research topic. All the authors have presented their research objectives to the board then the questionnaire got approval to conduct the study.

## Consent statement

The consent from all the participants involved in the study has been taken. A self-explanatory written statement was attached with the questionnaire for the participants and the similar questionnaire has been submitted to the university research board (REB).

## Data Availability

The underlying data related to the paper are available in figshare with the following citation and DOI. Figshare. Aggarwal, Megha, Badoni, Manish, Rawat, Babita (2024). Sociodemographic Analysis of Destination Selection Factors for Himalayan Hill Destinations. Dataset, https://doi.org/10.6084/m9.figshare.24936471.v2 ( Badoni et al., 2024). The data available were submitted by Megha Aggarwal licensed under CC BY 4.0. Complete details are-Socio-Demographic Analysis of Destination Selection Factors for Himalayan Hill Destinations © 2024 by Manish Badoni, Babita Rawat, and Megha Aggarwal licensed under CC BY 4.0. To view a copy of this license, visit http://creativecommons.org/licenses/by/4.0/.
