# Supplementary material for: Socio-demographic analysis of destination selection factors for Himalayan Hill destinations
Source: F1000Res. 2024 Sep 3;13:262. Originally published 2024 Apr 12. [Version 2] doi: 10.12688/f1000research.146873.2 (PMC11375401; doi:10.12688/f1000research.146873.2)
Supplement: Peer review history

# Reviewer report: Reviewer response for version 2

**DOI:** 10.5256/f1000research.171022.r320065

**Published:** 2024-09-04

**Authors:** Sunita Dwivedi

**Affiliations:** 1 Symbiosis Centre for Management Studies, Symbiosis International University, Pune, Maharashtra, India

Dear Editor

 I here by approve the paper

Is the work clearly and accurately presented and does it cite the current literature?

Yes

If applicable, is the statistical analysis and its interpretation appropriate?

Yes

Are all the source data underlying the results available to ensure full reproducibility?

Yes

Is the study design appropriate and is the work technically sound?

Yes

Are the conclusions drawn adequately supported by the results?

Yes

Are sufficient details of methods and analysis provided to allow replication by others?

Yes

Reviewer Expertise:

Marketing and Branding

I confirm that I have read this submission and believe that I have an appropriate level of expertise to confirm that it is of an acceptable scientific standard.

# Reviewer report: Reviewer response for version 2

**DOI:** 10.5256/f1000research.171022.r320066

**Published:** 2024-09-04

**Authors:** Devkant Kala

**Affiliations:** 1 School of Business, UPES, Dehradun, Uttarakhand, India

Thank you for incorporating the suggested changes in your revised manuscript.

 The flow, structure, and quality of the manuscript have improved now.

 There is only one comment:

 Please remove references from the research objectives.

Is the work clearly and accurately presented and does it cite the current literature?

Partly

If applicable, is the statistical analysis and its interpretation appropriate?

Yes

Are all the source data underlying the results available to ensure full reproducibility?

Partly

Is the study design appropriate and is the work technically sound?

Partly

Are the conclusions drawn adequately supported by the results?

Yes

Are sufficient details of methods and analysis provided to allow replication by others?

Partly

Reviewer Expertise:

Tourism & Hospitality and Consumer Behaviour

I confirm that I have read this submission and believe that I have an appropriate level of expertise to confirm that it is of an acceptable scientific standard.

## References

[1] : TOURISTS’ SATISFACTION AT TRIJUGINARAYAN, INDIA: AN IMPORTANCE-PERFORMANCE ANALYSIS. *https://dergipark.org.tr/en/pub/ahtr/issue/32311/359067#article_cite* .2015;

# Reviewer report: Reviewer response for version 1

**DOI:** 10.5256/f1000research.161000.r267431

**Published:** 2024-05-06

**Authors:** Sunita Dwivedi

**Affiliations:** 1 Symbiosis Centre for Management Studies, Symbiosis International University, Pune, Maharashtra, India

Dear Team F1000 Research,

 Thank you for providing this opportunity to review the paper. The theme is interesting and the study has used all novelty to the research area. My feedback is as below:

 The purpose of this study was to ascertain the correlation between various factors related to destination selection and sociodemographic characteristics of tourists for various destinations in the Himalayan region of Uttarakhand and Himachal Pradesh, India. Many destination factors, such as "value for money," "destination image," "beauty, culture, and heritage," "tour & travel connections," "value-added services," and "destination brand value," have been identified with the use of factor analysis techniques.

 Research has statistically validated the factors identified impacting young consumers destination selection preferences. The study concluded with the significant outcome for tour and travel industry to draw the marketing strategies for travelers. 

 I concur with the results and implication drafted by the authors, and recommend the paper for publication

 Thanks

Is the work clearly and accurately presented and does it cite the current literature?

Yes

If applicable, is the statistical analysis and its interpretation appropriate?

Yes

Are all the source data underlying the results available to ensure full reproducibility?

Yes

Is the study design appropriate and is the work technically sound?

Yes

Are the conclusions drawn adequately supported by the results?

Yes

Are sufficient details of methods and analysis provided to allow replication by others?

Yes

Reviewer Expertise:

Marketing and Branding

I confirm that I have read this submission and believe that I have an appropriate level of expertise to confirm that it is of an acceptable scientific standard.

# Reviewer report: Reviewer response for version 1

**DOI:** 10.5256/f1000research.161000.r267432

**Published:** 2024-04-24

**Authors:** Devkant Kala

**Affiliations:** 1 School of Business, UPES, Dehradun, Uttarakhand, India

Thank you for the opportunity to read this interesting study. The study has some potential. However, certain issues need to be addressed. The detailed observations are given below for your response:

**Abstract:**

 Refinement is needed, particularly in the methods and results sections.

 Introduction:

 The rationale of the study should be presented more effectively, with recent and relevant papers cited. It is noted that many cited studies are outdated; the author/s should include recent and relevant studies to build and present their arguments. Incorporating Indian studies would further strengthen and justify the arguments made. For example,[1] https://dergipark.org.tr/en/pub/ahtr/issue/32311/359067

**Hypotheses Development:**

 Hypotheses development is not sufficiently presented. Please provide relevant literature to support your hypotheses.

**Methods:**

 The author/s should provide references for previous studies from which items were adopted.

 Clear justification is needed for selecting Uttarakhand and Himachal Pradesh as study areas, as well as for choosing convenience sampling.

 “……. because Indian female respondents were reluctant to complete the survey, and the survey was performed by their spouses or other relatives.” Please refine this statement.

 What about the validity of the questionnaire?

 What was the language?

 Did you conduct a Pilot test before full-scale survey?

**Results:**

 Table 2: Item Description: Replace ‘You’ with ‘I’.

 Instead of ANOVA, I suggest the author/s use regression analysis between Factors 1-7 and Factor 8.

 The author/s should compare the results of the study with previous studies.

 The implications (Theoretical and managerial) are vague and need refinement.

 “Not every study has been completed without limitations,” – Please delete this portion of the statement.

Is the work clearly and accurately presented and does it cite the current literature?

Partly

If applicable, is the statistical analysis and its interpretation appropriate?

Yes

Are all the source data underlying the results available to ensure full reproducibility?

Partly

Is the study design appropriate and is the work technically sound?

Partly

Are the conclusions drawn adequately supported by the results?

Yes

Are sufficient details of methods and analysis provided to allow replication by others?

Partly

Reviewer Expertise:

Tourism & Hospitality and Consumer Behaviour

I confirm that I have read this submission and believe that I have an appropriate level of expertise to confirm that it is of an acceptable scientific standard, however I have significant reservations, as outlined above.

## References

[1] : ‘Thank you, God. You saved us' - examining tourists’ intention to visit religious destinations in the post COVID. *Current Issues in Tourism* .2021;24(22) : 10.1080/13683500.2021.1876643 3127-3133 10.1080/13683500.2021.1876643

# Response

**Published:** 2024-08-25

**Authors:** Manish Badoni

**Affiliations:** USHHM, Uttaranchal University, Dehradun, Uttarakhand, India

Dear Reviewer, Thank you for your suggestions. We have incorporated the major part as you suggested. However, one suggestion related to regression analysis has not been incorporated as we are developing a new paper using a similar analysis.
